# American Academy of Dental Science

**Published:** 1880-02

**Authors:** C. P. Wilson

**Affiliations:** Cor. Secretary.


### ARTICLE VIII.

### *American Academy of Dental Science.*

The American Academy of Dental Science, at the monthly meeting held in Boston, December 3rd, 1879, adopted the following resolutions:

It having pleased an all-wise Providence to call from the scene of his earthly labors and usefulness, our esteemed fellow-member, John Clough, M. D., of Woburn, Mass., therefore

*Resolved*, That the American Academy of Dental Science has heard with deep regret of the decease of our worthy friend and associate, Dr. John Clough, who, as one of the old practitioners of dentistry in Boston, for more than thirty years, faithfully upheld the honor of the profession.

*Resolved*, That in the death of Dr. Clough, the Academy has sustained the loss of one of its most zealous members, dental science an earnest and intelligent advocate, and the community an excellent and useful citizen.

*Resolved*, That we accord to his memory this heartfelt tribute of respect, and tender to his surviving family our condolence and sympathy in their heavy bereavment.

*Resolved*, That a copy of these resolutions, signed by the President, Vice President and Secretary, be presented to the family of the deceased, and a copy be entered upon the records of the Academy and published in the dental journals.

C. P. Wilson,
*Cor. Secretary.*

